# Rutin Isolated from *Chrozophora tinctoria* Enhances Bone Cell Proliferation and Ossification Markers

**DOI:** 10.1155/2018/5106469

**Published:** 2018-02-13

**Authors:** Ashraf B. Abdel-Naim, Abdullah A. Alghamdi, Mardi M. Algandaby, Fahad A. Al-Abbasi, Ahmed M. Al-Abd, Basma G. Eid, Hossam M. Abdallah, Ali M. El-Halawany

**Affiliations:** ^1^Medicinal Plants Research Group, Deanship of Scientific Research, King Abdulaziz University, Jeddah, Saudi Arabia; ^2^Department of Pharmacology and Toxicology, Faculty of Pharmacy, King Abdulaziz University, Jeddah, Saudi Arabia; ^3^Department of Biochemistry, Faculty of Science, King Abdulaziz University, Jeddah, Saudi Arabia; ^4^Department of Biological Sciences, Faculty of Science, King Abdulaziz University, Jeddah, Saudi Arabia; ^5^Pharmacology Department, Medical Division, National Research Centre, Giza, Egypt; ^6^Department of Natural Products, Faculty of Pharmacy, King Abdulaziz University, Jeddah, Saudi Arabia; ^7^Department of Pharmacognosy, Faculty of Pharmacy, Cairo University, Cairo, Egypt

## Abstract

Osteoporosis is a chronic disease in which the skeleton loses a weighty proportion of its mineralized mass and mechanical pliability. Currently available antiosteoporotic agents suffer adverse effects that include elevated risk of thrombosis and cancer. Phytochemicals may constitute a safer and effective option. In the current work, six flavonoids were obtained from *Chrozophora tinctoria* and identified as amentoflavone (1), apigenin-7-*O*-*β*-d-glucopyranoside (2), apigenin-7-*O*-6′′-*E*-*p*-coumaroyl-*β*-d-glucopyranoside (3), acacetin-7-*O*-*β*-d-[*α*-l-rhamnosyl(1→6)]3′′-*E*-*p*-coumaroyl glucopyranoside (4), apigenin-7-*O*-(6′′-*Z*-*p*-coumaroyl)-*β*-d-glucopyranoside (5), and rutin (6). An extensive review of the literature as well as NMR and mass spectral techniques was employed in order to elucidate the compound structures. Proliferation was enhanced in MCF7, MG-63, and SAOS-2 cells after exposure to subcytotoxic levels of the tested flavonoids. Rutin was chosen for subsequent studies in SAOS-2 cells. Rutin was not found to cause any alteration in the index of proliferation of these cells, when examining the cell cycle distribution by DNA flowcytometric analysis. Rutin was, however, found to increase osteocyte and osteoblast-related gene expression and lower the expression of RUNX suppressor and osteoclast genes. When examining the influence of rutin on vitamin D levels and the activity of alkaline phosphatase enzyme, it was found to enhance both, while decreasing acid phosphatase which is a marker of osteoporosis. Thus, rutin enhances proliferation and ossification markers in bone cells.

## 1. Introduction

Osteoporosis is a chronic disease in which the skeleton loses a weighty proportion of its mineralized mass and mechanical pliability [1]. It occurs when bone resorption surpasses bone formation, causing an imbalance [2]. As a result, the bones tend to become more fragile and more susceptible to fractures [3]. Studies have shown that 50% of women and 20% of men are likely to have a fracture resulting from osteoporosis during their lifetime [4]. Such fractures impose a heavy health and economic burden worldwide [5, 6]. The risk of developing osteoporosis has been shown to be directly linked to diet. Studies have reported that people eating healthy diets with a high fruit and vegetable content tend to have lower bone resorption than their counterparts eating poor diets rich in processed foods [7]. Pharmacological management of osteoporosis involves the use of bisphosphonates and estrogen replacement therapy. However, these medicines suffer adverse effects that may range from gastric irritation to increased thromboembolic and cancer risks [8–10]. Therefore, it is imperative that we look for safer and effective alternatives. In this regard, medicinal herbs and plant-derived molecules have gained wide acceptance by the public and scientific communities [11].

Flavonoids, which are widely found in fruit and vegetables, are bioactive polyphenols with anti-inflammatory and antioxidant properties. Bone health has been associated with the intake of flavonoids. Intake of flavonoids increases bone mass density (BMD) in the neck and spine and decreases bone resorption in perimenopausal women [12]. Moreover, catechins and flavanones were found to associate with markers of bone resorption negatively. At the hip and spine, anthocyanins were found to be strongly linked with BMD [13]. It has been postulated that the reduction of low-grade inflammation and oxidative stress by flavonoids is the hallmark of protecting bone loss. In addition, flavonoids are thought to promote the upregulation of signaling pathways that increase the activity of osteoblasts [14].

*Chrozophora tinctoria* (L.) A. Juss. has several other names including turnsole, Dyer's croton or giradol. It is an annual plant that belongs to the *Euphorbiaceae* family [15]. Experimentally, it exhibited antioxidant and wound healing effects [16, 17]. It exerts pronounced anti-inflammatory activities which involve inhibition of TNF-*α*, PGE_2_, IL-1*β*, and IL-6 [18]. Phytochemically, this plant is rich in biflavones, such as amentoflavone in addition to many flavonoids including apigenin, rutin, quercetin, and acacetin [15, 18–20]. The current work aimed at isolating, identifying, and assessing the activity of major flavonoids isolated from *C. tinctoria* on markers of ossification and proliferation of bone cells.

## 2. Materials and Methods

### 2.1. Material for Phytochemical Studies

UV IKON 940 spectrophotometer was used to measure UV spectra. A Bruker Apex III Fourier-transform ion cyclotron resonance (FTICR) mass spectrometer (Bruker Daltonics, Billerica, USA) including an Infinity™ cell and a 7.0 Tesla superconducting magnet (Bruker, Karlsruhe, Germany) was used to perform mass spectrometric studies. A Bruker DRX-600 MHz Ultrashield spectrometer (Bruker BioSpin, Billerica, MA, USA) was utilized to measure NMR spectra. Chromatographic separation of the active compounds was performed on Silica gel 60 (70-230 mesh, Merck, Darmstadt, Germany), Silica gel 100 C_18_-Reversed phase (0.04–0.063 mm, Merck, Darmstadt, Germany), and Sephadex LH-20 (Pharmacia Fine Chemicals Inc., Uppsala, Sweden). Monitoring of the isolation process was carried out on TLC plates with Silica gel 60 F_254_ (Merck, Darmstadt, Germany).

### 2.2. Plants Utilized in the Study

*Chrozophora tinctoria* (L.) A. Juss., Euphorbiaceae aerial parts were obtained from Al-Hadda road, Kingdom of Saudi Arabia (April 2015). These specimens were authenticated by Dr. Emad Al-Sharif, Department of Biology, King Abdulaziz University, Saudi Arabia. A specimen (reg. number CO-1080) was retained in the herbarium of the Department of Natural Products and Alternative Medicine, Saudi Arabia.

### 2.3. Phenolic Compound Extraction

The isolation process was performed as previously reported [18]. In brief, two kilograms of the aerial parts of *C. tinctoria* were dried and methanol was used as an extraction solvent till exhaustion to give a 150 g residue. The total extract was suspended in the least amount of water and extracted with chloroform leaving flavonoid-rich mother liquor that was separated using a Diaion HP-20 column starting with water up to 100% methanol to give three fractions (A–C). Fraction A was free from any phenolic compounds. Silica gel column chromatography was employed to separate fraction B (50 × 5 cm, 180 g). CHCl_3_ : MeOH was employed with gradient elution resulting in three fractions, I, II, and III. The first fraction (0.5 g) was separated on CC Sephadex LH-20 using the eluent MeOH to give compound 1 (50 mg). The second fraction (1.5 g) was subjected to chromatography with reversed phase Silica gel 100 C_18_–column and MeOH : H_2_O, 3 : 7 as an eluent to give compound 2 (40 mg). The third fraction (2 g) was repeatedly fractionated on Sephadex LH-20 using MeOH as an eluent; followed by CC on reversed phase Silica gel 100 C_18_ using a system of MeOH : water, 3 : 7; and finally purification was performed on HPLC using a Zorbax SB-C18 column (9.4 × 250 mm), flow rate 5 ml/min to give to compounds 3 (20 mg), 4 (35 mg), and 5 (45 mg). Fraction C was chromatographed on Sephadex LH-20 using MeOH as an eluent to give compound 6 (20 mg).

### 2.4. Chemical Compounds and Media

Sulfarhodamine B (SRB), RNAse-A enzyme, 17*β*-hydroxyestradiol, and propidium iodide (PI) were obtained from Sigma-Aldrich (St. Louis, MO, USA). Cell culture materials such as DMEM media, fetal bovine serum (FBS), McCoy's-5A media, and MEM media were obtained from Gibco™, Thermo Fisher Scientific (Waltham, MA, USA). The chemicals utilized in this study were of the highest available analytical grade.

### 2.5. Cell Culture

The cell lines used in this study were human breast adenocarcinoma cells (MCF-7 cell line), in addition to human osteosarcoma cell lines (SAOS-2 and MG-63). These estrogen-dependent cell lines were purchased from the VACCERA (Giza, Egypt). The cells were kept in DMEM, MEM, and McCoy's-5A media, respectively. Streptomycin (100 *μ*g/ml), penicillin (100 units/ml), and heat-inactivated FBS (10% *v*/*v*) were added to the media. Cells were kept in at 37°C and in humid conditions 5% (*v*/*v*) CO_2_ atmosphere.

### 2.6. Assessment of Cytotoxicity

The cytotoxic effects of the compounds obtained from *C. tincturia* were examined in MCF-7, SAOS-2, and MG-63 cells using SRB assay as described in the previous work [21]. Trypsin-EDTA (0.25% *w*/*v*) was utilized to briefly detach exponentially growing cells. Subsequently, cells were transferred to 96-well plates (10^3^ cells per well). The test compounds were applied for 72 h to the cells. The cells were then fixed using TCA (10% *w*/*v*) at 4°C for 1 h. Subsequently, the cells were washed three times and SRB solution (0.4% *w*/*v*) was added in a dark room for 10 min after which glacial acetic acid (1% *v*/*v*) was applied for a final wash. Cells were dried and the SRB-stained cells were dissolved by Tris-HCl (0.1 M). A microplate reader was utilized to record the color intensity at 540 nm.

### 2.7. Determination of Doubling Time Using the Proliferation Assay

SRB assay was employed to calculate the doubling time (as a measure for the proliferative effect) of MCF-7, SAOS-2, and MG-63 cells in the presence of and absence of incubation with the compounds isolated from *C. tincturia*. A subcytotoxic concentration (1 *μ*M) of the flavonoids obtained from *C. tincturia* was shortly applied to cells growing exponentially in media free of phenol red for 96 h. SRB solution was used to stain cells in order to carry out their quantification and to calculate doubling time using the best fit linear regression analysis curve [22].

### 2.8. Cell Cycle Distribution Study

In order to determine the effects of rutin on the cell cycle distribution, 1 *μ*M rutin was applied to SAOS-2 cells for 48 h and a comparison was made relative to 0.1 *μ*M estradiol. Trypsin was used to detach and collect the cells, after which PBS was added to wash the cells twice at 4°C. The cells were then resuspended in PBS (0.5 ml). Ethanol (ice-cold 70% *v*/*v*) (2 ml) was applied while shaking. This was followed by an incubation of the cells for 1 h at 4°C to allow fixation to occur. Cells were then analyzed, washed, and resuspended in PBS (1 ml) containing 50 *μ*g/ml of RNAase-A and 10 *μ*g/ml of PI. The cells were kept in the dark (20°C) for 20 minutes and the DNA contents were determined. After being passed into an ACEA Novocyte™ flow cytometer (ACEA Biosciences Inc., San Diego, CA, USA), the cells were analyzed using a FL2 signal detector (*λ*ex/em 535/617 nm) for PI positive events. Phases of the cell cycle were determined by ACEA NovoExpress™ software (ACEA Biosciences Inc., San Diego, CA, USA) for every sample (12,000 events per sample) subsequent to defining cell fragment-free fluorescent gate. Cells in the supra-G_2_/M phase were identified using ungated events. Total cells in S- and G_2_/M phases were divided by cells in G_0_/G_1_ phases in order to calculate the proliferation index.

### 2.9. Gene Array Studies

Expression of osteogenic- and osteolytic-related genes was studied by exposing the cells (1 × 10^6^) to either 1 *μ*M rutin or 0.1 *μ*M estradiol for 48 h. The latter served as a positive control. RNeasy Mini Kit® (Qiagen Inc., Valencia, CA, USA) was used for RNA extraction. cDNA was obtained by reverse transcription with a cDNA Reverse Transcription Kit (Applied Biosystems, Foster City, CA). PCR was performed in real time on the cDNA via GeneQuery™ Human Osteogenic Differentiation qPCR Array (Science Cell Research Laboratories Inc., Carlsbad, CA, USA) according to the manufacturer's instructions [23]; *β*-actin was selected as the housekeeping gene. The formula: 2^−ΔΔCq^ was employed to detect the normalized change in the gene expression of the studied genes after treatment with rutin and estradiol.

### 2.10. Osteoporosis Marker Assessment

The effects of rutin on ossification markers in the SAOS-2 cell line were determined by the application of 1 *μ*M rutin or 0.1 *μ*M estradiol to 1 × 10^6^ cells for 48 h. The latter served as a positive control. After collecting the media, assays of vitamin D, alkaline phosphatase (ALP), osteocalcin (OC), and acid phosphatase (ACP) were performed. A direct HTS-ready colorimetric kit (Abcam, Cambridge, UK) was used to determine ALP as well as tartrate-resistant ACP [24]. Uscan® immunoassay ELISA Kit (Life Science Inc., Wuhan, China) was utilized to detect osteocalcin [25]. Human Total 25-OH Vitamin D IVD ELISA Kit (R&D Systems, Inc., Minneapolis, MN, USA) was used to detect vitamin D in its active form.

### 2.11. Statistical Analysis

Data are presented as mean ± SD. Analysis of variance (ANOVA) and Tukey's post hoc test were used to calculate statistical significance by SPSS® for Windows, version 17.0.0. *p* < 0.05 was regarded as being statistically significant.

## 3. Results and Discussion

**Figure 1:**
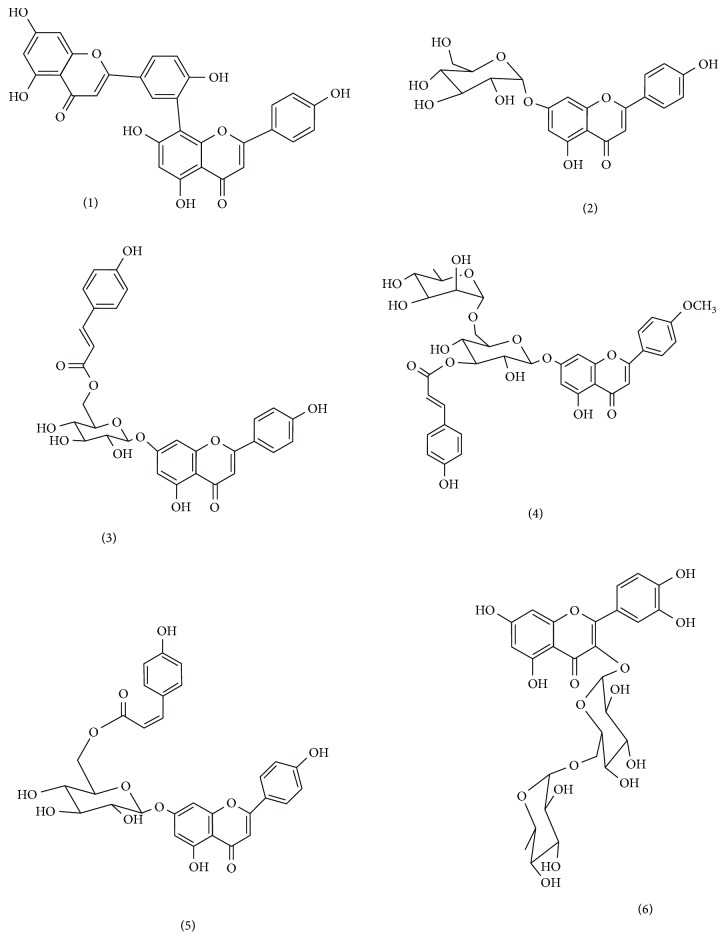
Isolated compounds from *Chrozophora tincturia*.

There is an immense need for the development of novel drugs to treat osteoporosis which are devoid of potentially life-threatening side effects, namely, stroke and carcinogenesis [8, 26]. We have previously found that the phenolic compound paradol, isolated from *Aframomum meleguea* seeds, showed proliferative effects in bone cells [27]. Flavonoids have been shown by numerous studies to prevent bone loss [12, 28–31]. Since *C. tinctoria* is rich in flavonoids as apigenin, rutin, quercetin, and acacetin [18, 20], we examined the effects of these compounds on proliferation and ossification markers. Mechanistically, the plant has been shown to impede several pathologic processes leading to osteoporosis as oxidative stress and inflammation [18]. Our study focused on isolating and determining the activity of its flavonoids on bone cell proliferation and ossification markers. Six metabolites were isolated from the *C. tinctoria* after phytochemical analysis (Figure 1). Cochromatography with authentic samples was used to identify these compounds, in addition to referring to spectral data from the literature. The identified compounds were amentoflavone (1) [32, 33], apigenin-7-*O*-*β*-d-glucopyranoside (2) [34], apigenin-7-*O*-6′′-*E*-*p*-coumaroyl-*β*-d-glucopyranoside (3) [35], acacetin-7-*O*- *β*-d-[*α*-l-rhamnosyl(1→6)]3′′-*E*-*p*-coumaroyl glucopyranoside (4) [18], apigenin-7-*O*-(6′′-*Z*-*p*-coumaroyl)-*β*-d-glucopyranoside (5) [18], and rutin (6) [34].

The cytotoxicity of these compounds was studied using the SRB viability assay in various estrogenic cell lines, namely, MCF-7, SAOS-2, and MG-63 cells. We have previously used these cell lines to study the effects of paradol on *A. meleguea* seeds, where we noted an accelerated proliferation [27]. In the present study, when looking at the effects on MCF-7 cells, the compounds showed a maximum of 20% alteration in the viability of cells, after being applied to the compounds for 72 h in 10 *μ*M concentrations. Exposing the cells to a higher concentration (100 *μ*M) of amentoflavone for 72 h (1) lead to a 78.8% viability of the control untreated cells (Table 1). As in the MCF-7 cells, exposure of SAOS-2 cells to the compounds at 10 *μ*M concentration for 72 h showed a maximum of 20% change in viability. However, increasing the concentration of the compounds 1, 3, 5, and 6 to 100 *μ*M induced a viability drop down to 70.1%, 66.2%, 77.1%, and 76.2% of the untreated control cells, respectively (Table 1). Interestingly, MG-63 cells were the most affected by the compounds under investigation. The addition of the compounds under investigation (0.1 *μ*M) to MG-63 cells for 72 h had no more than a 20% change in the viability of the cells. However, exposure to 1 *μ*M of 1 and 5 reduced cell viability to 74.9% and 78.1% of the control cells, respectively. The addition of a greater concentration (10 *μ*M) of all flavonoids under investigation induced 20–40% killing effect at 72 h of exposure. Further exposure to 100 *μ*M of all compounds under investigation dropped cell viability of MG-63 cells to 40–70% of the control untreated cells (Table 1**)**.

It has been previously reported that several flavonoids and related phytochemicals interact with estrogen receptors [36, 37]. The influence of a subcytotoxic concentration (1 *μ*M) from all compounds under investigation on doubling times of MCF-7, SAOS-2, and MG-63 cells was tested. A concentration of 0.1 *μ*M E_2_ served as a positive control. Compounds 1, 3, and 6 caused a significant lowering of the doubling times of MCF-7 cells from 16.6 ± 1.4 h to 9.3 ± 0.2 h, 8.6 ± 0.4 h, and 7.2 ± 0.5 h, respectively. The addition of E_2_ to MCF-7 cells resulted in a lowering of the doubling time to 8.5 ± 0.4 h (Table 2). When studying the cell lines derived from bone osteosarcoma, compounds 1, 3, 4, 5, and 6 caused a decrease in the doubling time of SAOS-2 cells from 49.3 ± 4.1 h to 26.6 ± 1.4 h, 14.7 ± 0.7 h, 29.0 ± 2.9 h, 29.4 ± 3.5 h, and 15.7 ± 0.2 h, respectively. In SAOS-2 cells, it was noted that E_2_ decreased the doubling time to 20.1 ± 1.2 h (Table 2). In addition, compounds 1, 3, 5, and 6 lowered the MG-63 cells doubling time from 36.8 ± 2.2 h to 20.9 ± 0.3 h, 21.0 ± 0.3 h, 23.5 ± 1.1 h, and 20.8 ± 0.5 h, respectively, in comparison to 23.1 ± 0.1 h by E_2_ (Table 2). It is worth mentioning that some flavonoids might be equipotent or ever more potent than E_2_ which might be attributed to the higher concentrations used (more than 10-fold). When comparing all tested flavonoids, rutin (6) showed promising proliferative properties with minimal expected mutagenic influence. This is in line with reports by Hyun et al. regarding its osteoclast activating properties [38]. Additionally, the selection of SAOS-2 cells for future studies was based upon their low viability drop when treated with the flavonoids being studied.

**Figure 2:**
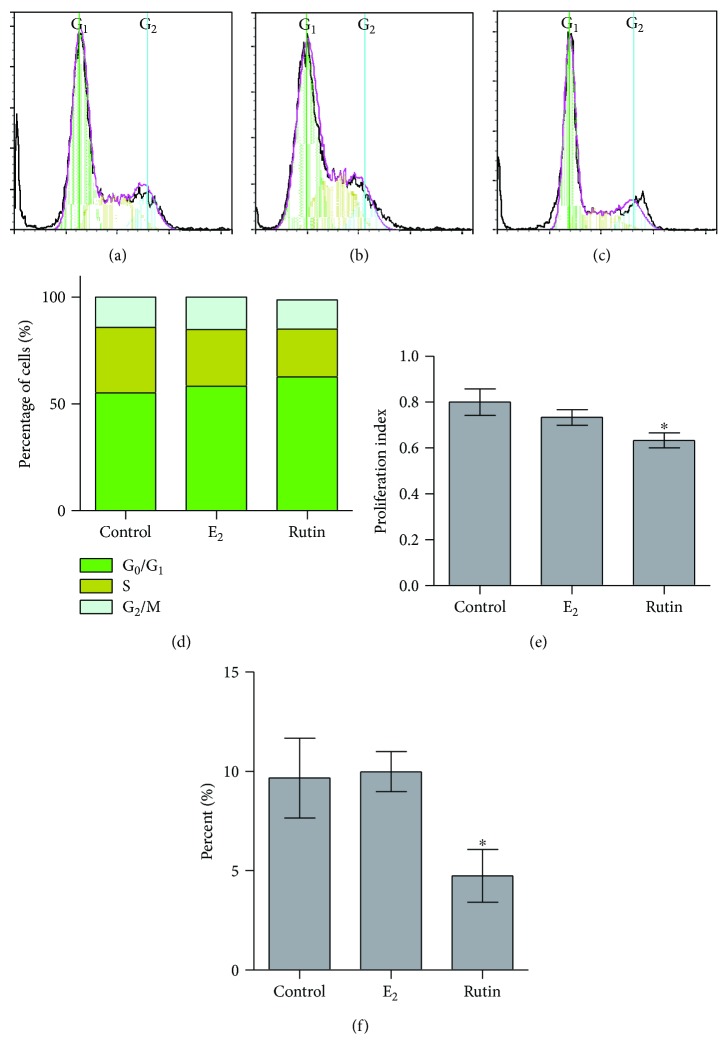
Effect of rutin on the cell cycle distribution of SAOS-2 cells. Cells were exposed to rutin (1 *μ*M) for 48 h (b) and compared to control untreated cells (a) and E_2_- (0.1 *μ*M) treated cells (c). Cell cycle distribution was determined using DNA cytometry analysis and different cell phases were plotted (d) as percentage of total events. Proliferation index was calculated and plotted (e). Supra-G_2_/M cell population was plotted as percent of total events (f). Data are presented as mean ± SD; *n* = 3. ^∗^Significantly different from the control untreated cells; *p* < 0.05.

In this study, the flavonoids obtained from *C. tinctoria* displayed clear proliferative effects in all the three studied cell lines. Although several studies have reported that estrogen along with various estrogen metabolites possesses proliferative properties, this was limited by the fact that they cause mutagenicity [22, 39]. Therefore, it was of importance to record the impact of rutin on the distribution of the phases of cell cycle in relation to the progression of the cell cycle flow cytometry to determine DNA content. Treatment of SAOS-2 cells with 1 *μ*M rutin for 48 h was carried out. Exposing the cells to 0.1 *μ*M E_2_ served as a positive control. Interestingly, rutin produced a significant increase of cells in G_0_/G_1_ from 55.2 ± 2.4% to 62.6 ± 1.1% with a reciprocal decrease for cells in S-phase from 30.7 ± 2.9% to 22.5 ± 1.0%. G_2_/M phase was not affected by treatment with rutin (Figures 2(a), 2(c), and 2 (d)). However, 0.1 *μ*M E_2_ produced a significant drop of cells in the S-phase (30.7 ± 2.9% to 26.5 ± 0.9%). Treating SAOS-2 cells with E_2_ caused no significant changes of cells in G_0_/G1 or S-phases (Figures 2(a), 2(b), and 2(d)). When studying the proliferating cell fraction balance in SAOS-2 cells, rutin significantly decreased the proliferation index; on the other hand, treatment with E_2_ did not alter their proliferation index (Figure 2(e)). In a previous study in our laboratory, paradol showed no influence on the proliferation index of SAOS-2 cells [27]. Our current data are in alignment with our studies on SAOS-2 cells where 1 *μ*M rutin was found to decrease the doubling time. Yet, we tested the nature of the rutin proliferative effect in SAOS-2; accumulation of cells in the supra-G_2_ compartment (multiploidy phase) might indicate uncontrolled cell proliferation. Interestingly, after SAOS-2 exposure to rutin, cells in supra-G_2_ phase were much lower than control cells (Figure 2(f)). This indicates a punctuated cell division without cell accumulation in multiploidy phase [40]. This gains support by the known chemopreventive and anticarcinogenic properties of rutin [41–43].

**Figure 3:**
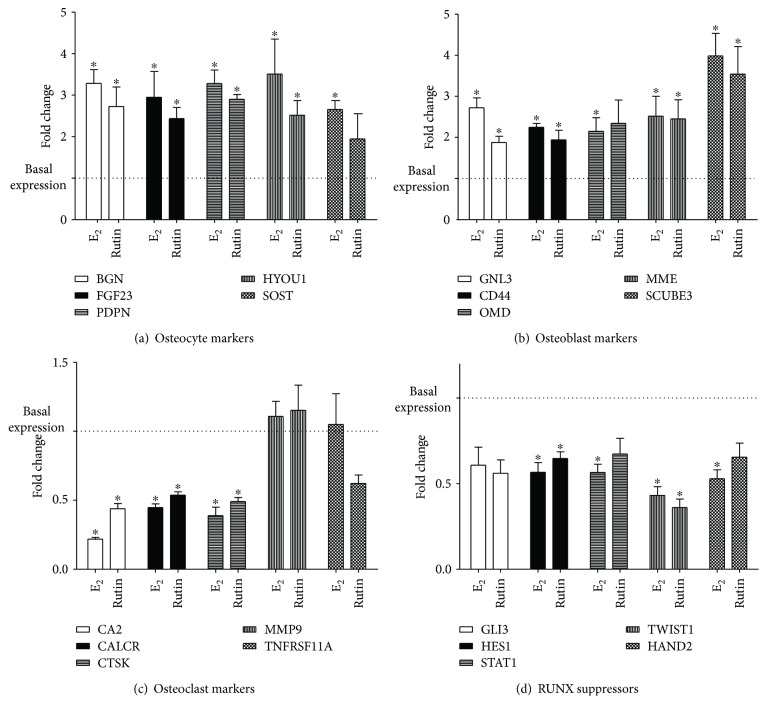
Effect of rutin on mRNA expression of some ossification related genes in SAOS-2 cell line. Cells were incubated with rutin (1 *μ*M) or E_2_ (0.1 *μ*M) for 48 h. Total RNA was extracted and subjected to RT-PCR. Data were normalized to *β*-actin; fold changes were calculated and expressed as mean ± SD; *n* = 3. ^∗^Significantly different from the control untreated cells; *p* < 0.05.

Several studies have reported that targeting ossification is a more fruitful approach than targeting proliferation, when looking at developing new drug targets to treat osteoporosis. [44, 45]. The effects of rutin on osteogenic marker gene expression were quantitatively studied using RT-PCR gene array battery kit that comprises various osteogenic gene families. These include osteocyte, osteoclast, and osteoblast activity markers, as well as RUNX suppressor genes. When studying the osteocyte activity markers (*BGN*, *FGF23*, *PDPN*, *HYOU1*, and *SOST*), rutin and E_2_ caused an increase in expression of all the tested osteocyte activity gene markers by 1.9- to 2.9-folds and 2.7- to 3.5-folds, respectively (Figure 3(a)). The role of osteocytes in calcium and phosphate homeostasis has been previously reported. Osteocytes cloned with BGN caused acceleration of osteoblast differentiation in vitro and an increase in the area of lamellar bone-like matrices in vivo [46]. Phosphate levels are known to decline due to the presence of the FGF23 protein [47]. Dividing osteocytes are known to express PDPN, which is regarded as a marker of activity [48]. They also secrete SOST causing a negative effect on the formation of bones [49]. Also, rutin increased the expression level of *GNL3*, *CD44*, *MME*, and *SCUBE3* osteoblast activity markers, by 1.8 ± 0.2-, 1.9 ± 0.3-, 2.5 ± 0.7-, and 3.5 ± 0.9-folds, respectively. *OMD* expression after rutin treatment was not changed. The five tested gene markers of osteoblast activity were augmented by 2.1- to 4.0-folds after the application of E_2_ (Figure 3(b)). Compared to our previous work on paradol [27], rutin significantly increased HYOU1 but not SOST. Further, rutin could significantly enhance expression of the osteoblast marker CD44 while paradol failed to exert a similar effect. With respect to osteoclast activity markers, both rutin and E_2_ downregulated *CA2*, *CALCR*, and *CTSK* expression to 0.4- to 0.5-folds and 0.2- to 0.4-folds, respectively. The expression of *MMP9 and TNFRSF11A* genes was unaltered after treatment with either rutin or E_2_ (Figure 3(c)). These findings confirm the potential favorable effects of rutin. This is further supported by studies in the bone marrow that showed rutin inhibits osteoclastogenesis [50]. When studying the *RUNX* suppressor gene family (*GLB*, *HES1*, *STAT1*, *TWIST1*, and *HAND2*), rutin downregulated the expression of *HES1*and *TWIST1* mRNA to 0.7 ± 0.1- and 0.4 ± 0.07-folds of the control cells, respectively. On the other hand, E_2_ was found to suppress *HES1*, *STAT1*, *TWIST1*, and *HAND2* gene expression to 0.4- to 0.6-folds of control (Figure 3(d)). Studies have reported that RUNX regulates RANKL leading to the maturation and differentiation of osteoblasts [51]. The lowered expression of *HES1*and *TWIST1* (RUNX suppressors) by rutin supports its osteogenic effects. All these data are in alignment with our previous publication on paradol [27]. However, rutin showed superior activities with regard to suppressing *CALCR* expression.

**Figure 4:**
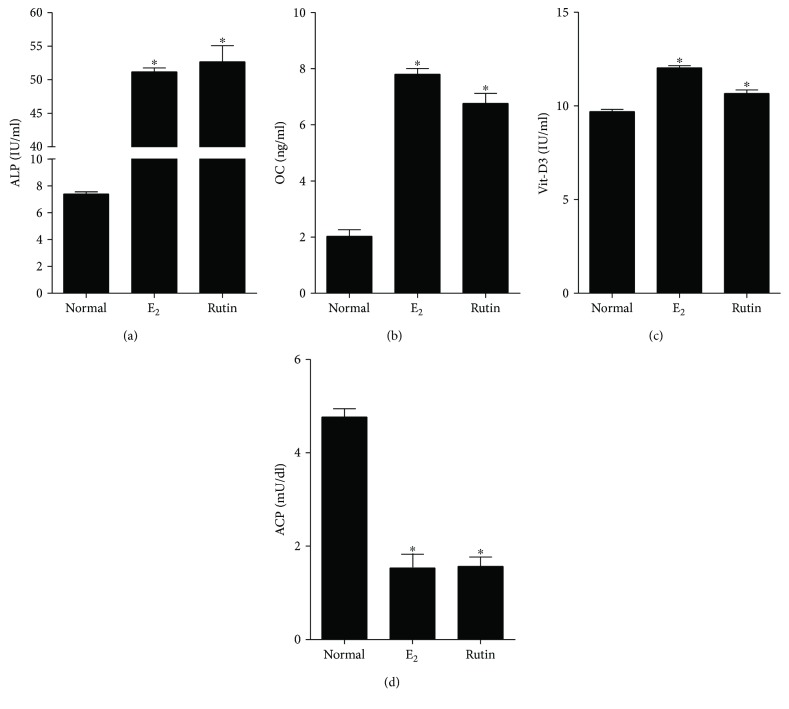
Biochemical assessment for antiosteoporosis effect of rutin *in vitro.* SAOS-2 cells were treated with rutin (1 *μ*M) and E_2_ (0.1 *μ*M) for 48 h and compared to the control untreated cells. Biochemical assessment of osteoporosis was evaluated by measuring alkaline phosphatase (a), osteocalcin (b), vitamin D3 (c), and acid phosphatase (d). Data are presented as mean ± SD; *n* = 3. ^∗^Significantly different from the control untreated cells; *p* < 0.05.

In the present study, we determined the effects of treating SAOS-2 cells with 1 *μ*M rutin for 48 h on the levels of four essential osteoporosis-related markers. The results were compared with treatment of the cells with 0.1 *μ*M E_2_ as a positive control. Rutin produced a significant increase in the activity/concentrations of all ossification markers, ALP enzyme, OCN hormone, and active Vit-D3 concentration, by 7.1-, 3.4-, and 1.1-folds, respectively. Similarly, E_2_ increased the activity/concentrations of ALP enzyme, OCN hormone, and active Vit-D3 concentration, by 6.9-, 3.9-, and 1.2-folds, respectively (Figures 4(a)–4(c)). On the other hand, rutin produced a statistically significant drop in ACP enzyme activity (bone resorption marker) from 4.7 ± 0.4 IU/ml to 1.6 ± 0.4 IU/ml compared to 1.5 ± 0.4 IU/ml for E_2_-treated cells (Figure 4(d)). The biochemical markers chosen in this study are well documented in the literature as reliable markers having antiosteoporotic properties. [52]. In conclusion, the analysis of *C. tinctoria* extract led to the isolation of various flavonoids showing antiosteoporosis influence. Rutin was especially promising as it showed ossification in bone cells as well as possessing punctuate proliferative activity with minimal influence to cell cycle distribution. In other words, rutin might be further studied as a potential antiosteoprotic agent with minimal expected mutagenic effects.

## Figures and Tables

**Table 1:** Cytotoxicity assessment of compounds isolated from *C. tincturia.*

	Cpd	Percent viability				
		0.01 *μ*M	0.1 *μ*M	1 *μ*M	10 *μ*M	100 *μ*M
MCF-7	1	95.8 ± 1.6	91.5 ± 3.4	88.6 ± 3.3	84.8^∗^ ± 1.6	78.8^∗^ ± 0.3
	2	99.4 ± 1.2	94.8 ± 3.3	91.1 ± 3.2	90.3 ± 2.4	85.9^∗^ ± 1.6
	3	99.1 ± 0.3	95.1 ± 3.4	94.0 ± 2.5	92.8 ± 1.7	90.5^∗^ ± 0.3
	4	99.8 ± 0.5	96.0 ± 0.5	94.5 ± 3.1	91.1 ± 2.4	87.9^∗^ ± 0.6
	5	98.3 ± 1.2	91.8 ± 2.7	88.9 ± 2.8	86.9^∗^ ± 1.7	80.2^∗^ ± 1.2
	6	97.5 ± 1.5	93.2 ± 3.8	90.4 ± 3.4	88.2^∗^ ± 2.1	80.3^∗^ ± 1.7

SAOS-2	1	98.9 ± 0.8	95.1 ± 0.9	92.1 ± 0.9	80.4^∗^ ± 0.9	70.1^∗^ ± 0.2
	2	99.2 ± 0.6	97.4 ± 2.0	94.5 ± 1.3	92.8 ± 1.6	89.3^∗^ ± 1.5
	3	97.4 ± 1.0	96.8 ± 0.9	91.9 ± 1.7	84.7 ± 3.4	66.2^∗^ ± 1.2
	4	98.4 ± 0.7	95.8 ± 0.8	92.7 ± 0.4	90.1 ± 0.9	84.7^∗^ ± 1.0
	5	97.0 ± 1.5	92.9 ± 0.8	90.9 ± 0.5	85.4^∗^ ± 0.5	77.1^∗^ ± 0.5
	6	96.6 ± 0.8	93.6 ± 0.7	92.6 ± 1.9	88.5^∗^ ± 1.2	76.2^∗^ ± 0.9

MG-63	1	88.2 ± 0.1	82.3 ± 1.2	74.9^∗^ ± 2.0	61.3^∗^ ± 0.4	40.4^∗^ ± 0.5
	2	95.9 ± 2.7	88.8 ± 2.0	81.1^∗^ ± 0.3	74.9^∗^ ± 1.4	70.9^∗^ ± 0.6
	3	96.7 ± 2.0	92.2 ± 1.6	87.4 ± 1.2	80.8^∗^ ± 0.2	64.3^∗^ ± 1.1
	4	98.1 ± 0.4	93.2 ± 1.3	87.2^∗^ ± 0.8	79.8^∗^ ± 2.0	60.2^∗^ ± 1.7
	5	93.1 ± 0.9	83.4 ± 1.2	78.1^∗^ ± 0.8	67.7^∗^ ± 1.1	54.7^∗^ ± 2.1
	6	91.1 ± 0.7	86.2 ± 0.8	82.2 ± 1.2	73.0^∗^ ± 1.2	63.4^∗^ ± 0.4

Cells were treated with test compounds for 72 h and viability was determined using SRB assay. Data are expressed as mean ± SD; *n* = 6. ^∗^Significantly different from control untreated cells (*p* < 0.05).

**Table 2:** Proliferative effects of compounds isolated from *C. tincturia*: doubling time assessment.

	MCF-7	SAOS2	MG-63
Control	16.6 ± 1.4	49.3 ± 4.1	36.8 ± 2.2
1	9.3^∗^ ± 0.2	26.3^∗^ ± 1.4	20.9^∗^ ± 0.3
2	14.8 + 1.7	35.7 ± 3.4	27.5 ± 2.1
3	8.6^∗^ ± 0.4	14.7^∗^ ± 0.7	21.0^∗^ ± 0.3
4	11.8 ± 3.6	29.0^∗^ ± 2.9	25.8^∗^ ± 3.5
5	13.4 ± 3.2	29.4^∗^ ± 3.5	23.5^∗^ ± 1.1
6	7.2^∗^ ± 0.5	15.7^∗^ ± 0.2	20.8^∗^ ± 0.5
E_2_	8.5^∗^ ± 0.4	20.1^∗^ ± 1.2	23.1^∗^ ± 0.1

Cells were treated with test compounds (1 *μ*M) for up to 96 h and viability was determined using SRB assay. Doubling times were calculated and compared to control untreated cells and E_2_ (0.1 *μ*M) treated cells (positive control). Data are expressed as mean ± SD; *n* = 6. ^∗^Significantly different from the corresponding untreated cells; *p* < 0.05.
